# A White Death Among the Ranks. Tuberculosis in the Austro-Hungarian Army, 1882–1914

**DOI:** 10.1093/shm/hkae095

**Published:** 2025-05-12

**Authors:** Jan Błachnio

**Affiliations:** University of Warsaw, Faculty of History, Krakowskie Przedmieście 26/28,00-927 Warsaw, Poland

**Keywords:** Austria-Hungary, military, tuberculosis, treatment, morbidity, mortality

## Abstract

This article is devoted to the incidence of tuberculosis in the Austro-Hungarian Imperial-Royal Army between the years 1882 and 1914, with a special focus on the region of Cisleithania. The first part of the article discusses the organisation of the Austro-Hungarian armed forces, drawing attention to those features of the organisation that may prove informative when researching the health status of the wider population as a whole. The question of the extent of the threat of tuberculosis in Austria-Hungary is then addressed, which shall allow us to move on to the incidence of the disease in the Imperial Royal Army. Drawing on archivally collated statistics, I consider the morbidity-mortality curves and the susceptibility to the disease of the soldiers of each army type. The next part of the paper considers a possible crossover between the incidence of tuberculosis in civilian society and in the army, using the example of selected regiments billeted in Cisleithania. The last part of the article shall discuss the preventive measures taken by military authorities, such as dismissing those struck down with the disease from active service and the implementation of a more rigid hygienic regime.

During wars fought by revolutionary France, military forces were organised through general conscription (*levée en masse*), a phenomenon which became the norm in the nineteenth century across continental European countries. In theory, at least, armed forces reflect a perfect mirror image of civilian society. Although such a reflection until recent times would have been devoid of a gender structure, it would have obtained for social characteristics such as class, religion and ethnic structures, educational levels, living standards and, thus, physical and health conditions. It is therefore worth contemplating whether this mirror metaphor could be applied to research on public health in the late nineteenth and early twentieth centuries, when soldiers enjoyed free medical care, and especially with reference to non-sexually transmitted diseases, including tuberculosis.

In the nineteenth and the first half of the twentieth centuries, tuberculosis was one of the most widespread social ailments. Its progression was caused by the TB bacillus (*Mycobacterium tuberculosis*, also known as Koch’s bacillus, after Robert Koch, the researcher who discovered it) and bovine mycobacterium (*Mycobacterium bovis*) whose main carrier was *Bos Taurus* or domesticated cattle. Although human TB is usually spread by droplet transmission for cattle, human infection with bovine TB mostly happens through the consumption of infected meat or milk, or due to lack of hygiene when working with domesticated bovines. The disease, which develops in approximately 10% of those infected, may affect various body organs.^1^

The most common forms of TB include lung TB, skeletal TB (due to the area it commonly affects—the disease may also infect the central nervous system) and skin TB. Furthermore, mycobacteria may also impact the excretory system, abdominal cavity or sexual organs among other parts of the body. Another TB form common in Austria-Hungary at the turn of the nineteenth and twentieth centuries was scrofulosis, which affected the lymph nodes.^2^

To date, there has been no attempt to determine the actual extent of tuberculosis epidemics in the periphery of Central-East Europe at the turn of the nineteenth and twentieth centuries. What is more, research has failed to establish a correlation between tuberculosis in the Austro-Hungarian armed forces in general and the civilian population, as well as tuberculosis in what was also known as the Common Army, or the Imperial and Royal Army. And inevitably, given these aforementioned oversights, no research has been carried out as to the evolution of opinion within the Austro-Hungarian army itself regarding the incidence of tuberculosis and the available options for its eradication.

What follows represents an attempt to present the incidence of tuberculosis (also known as consumption, or colloquially ‘the White Death’) in the Austrian part of the Austro-Hungarian armed forces from 1882 until 1914, and the outbreak of the First World War. On the other hand, the geographical framework of the study, in addition to the dual organisation of the Habsburg state, is influenced by the way in which the recruitment areas of infantry regiments in the Kingdom of Hungary were delimited, the boundaries of which were often kept separate from the shape of the territorial administration units (it was not uncommon for parts of two counties to be combined within a recruitment district). This state of affairs was prompted by the Hungarian authorities’ desire to ensure that soldiers of Hungarian nationality outnumbered those belonging to other ethnic groups in the regiments recruited in the Transleithania.^3^ When combined with the fact that epidemiological data was published in both parts of the dual monarchy (the Hungarians collected data for counties - vármegye, i.e. areas larger than counties), this means that any collation of military and civilian spheres poses something of a challenge.

And so, this article will not only signal research gaps, but will also indicate possible ways of filling such gaps by drawing attention, among other things, to the discrepancy between civilian and military statistics (caused by the accuracy of military screening tests and the detection of inactive tuberculosis). The perspective adopted here centres the approach on a subject characteristic for modern military history and the history of medicine, thus allowing a fuller picture to be grasped. Indeed, what follows seeks to verify whether there was an evolution in the attitude of the Austro-Hungarian military authorities towards the problem of tuberculosis between 1882 and 1914. Furthermore, the article points to the lack of simple translatability of military medical statistics to the health situation of civilians.

## A Taxonomical Research Overview

The body of work written on the historical role of tuberculosis is extensive, and multifaceted, and includes topics such as the cross-sectional history of tuberculosis (Robert Y. Keers^4^, Thomas Dormandy^5^, Frank Ryan^6^, Flurin Condrau and Michael Worboys^7^), the treatment of the disease (Francis B. Smith^8^, Thomas M. Daniel^9^), and the threat to the human population from bovine tuberculosis (Keir Waddington^10^). In addition, specific issues of the incidence and treatment of tuberculosis have been addressed by researchers such as Christoph Gradmann^11^ (a study of experimental research into the discovery of tuberculin), Donald S. Burke^12^ (a discussion of the circumstances surrounding Koch’s announcement of the discovery of tuberculin as a putative vaccine for tuberculosis); and John A. Odell^13^ (thoracic surgery used to treat tuberculosis). Relatively few studies address the social role of tuberculosis and systemic attempts to combat it in central and eastern Europe at the turn of the 20th century.

The problem of Koch’s bacillus, a causative agent of tuberculosis in the armed forces, has also received noticeably less attention from researchers, and is most often discussed in the context of military sanitation during conflict. In the setting of Austria-Hungary, special mention should be made here of the research of Brigitte Biwald, who devoted her doctoral dissertation to examining the imperial sanitary services during the First World War.^14^ Against the backdrop of the concentration of research interest in the incidence of disease during this armed conflict, James D. Mancuso’s article^15^ stands out, with its consideration of the screening tests and changes in the epidemiology of tuberculosis in the US Army from 1885 to the present day, with a particular emphasis on disease contraction during military campaigns.

Addressing the issue of the peacetime prevalence of various forms of tuberculosis in the Imperial and Royal Army of Austria-Hungary, also known as the joint or Common Army, required the adoption of research viewpoints concerning both the social role of tuberculosis, (or infectious diseases more broadly) and interaction between soldiers and civilians. A multifaceted study of the social history of tuberculosis in those Habsburg Crown Lands that make up Austria today and in the First Austrian Republic was conducted by Elisabeth Dietrich-Daum.^16^ Her findings on how attempts to treat the disease or Koch’s bacillus-induced mortality in the interwar army, known as the *Bundesheer*, would provide the basis for future research conceptualisations. Also relevant for research into the problem of tuberculosis in the Habsburg army were the works of Michał Baczkowski, set in the context of social history and modern military history, who analysed the modernising influence of the Austro-Hungarian army on the civilisational development of Galicia.^17^ Although Baczkowski’s studies focus on the problems of the economy and infrastructural development, and marginalising medical and sanitary issues, they do look at social history through the prism of military sources. Studies devoted to the place of the armed forces in late 19th- and early 20th-century societies were also important for the final shape of this article. The works of Johann Christoph Allmeyer-Beck^18^ (which deal with the place of the Habsburg army in the state and society) and the studies by Simon Giles Higgens^19^ and Edward M. Speirs^20^ devoted to the British Army should be mentioned here.

In terms of research methods, on the other hand, the studies of Alexander Mercer^21^, who focused his research on the shift in the main cause of English deaths from infectious to chronic diseases in the eighteenth and nineteenth centuries, using medical statistics would prove applicable in non-British contexts.

## The Methodological Approach and the Source Database

The sources on which this paper is based derive from the Austro-Hungarian civilian and military almanacs containing information on public health in the Habsburg State. The civilian data on infectious diseases was collected by the Ministry of the Interior of the Habsburg Crown Lands (*k.k. Ministerium des Innern*), as well as that of the Kingdom of Hungary (*Belügyminisztérium*).^22^ For the purposes of the research presented here, the records of the 14th Department (*Abteilung*) of the Ministry of War constitute a worthy supplement to the source database, in that they concern TB diagnoses in particular, as well as regulations concerning soldier examinations and procedures in the case of an incidence of the disease.

In 1882-1914, in Hungary, a decision was made not to publicise the number of cases of disease. And so, in Cisleithania only the number of cases which had completed hospitalisation was provided (including lung TB and scrofulosis-induced cases), with the number including hospital deaths as of 1884. Furthermore, in 1896 in Cisleithania, and in Hungary from 1898, general mycobacteria-induced deaths statistics were published. In 1910, the scrofulosis statistics were changed to include types of tuberculosis other than lung TB.^23^ The Cisleithanian statistics would prove to be more accurate as they showed the situation in particular administrative districts, while the Hungarian data failed to go lower than county level administratively. Austro-Hungarian population data were collected at ten-year intervals and date from 1880, 1890, 1900 and 1910.^24^

The military medical statistics for the period 1870–1912 were compiled from a survey covering the total population of the joint army, i.e. active service soldiers. The size of the sample varied over time, ranging from around 276,000 men in 1882 to nearly 450,000 in 1914, due to the fact that ranks of active service soldiers in the Austro-Hungarian population was swelled considerably over the same period.^25^ The statistics show the incidence of disease in the corps districts and individual types of armed forces, taking into account the number of soldiers and reservists discharged or placed on leave due to illness. The typology of Austro-Hungarian military statistics is shown in Table 1.

**Table 1.** Typology of Austro-Hungarian military statistics

Published military statistics					
Title	*Militär-statistisches Jahrbuch*		*Statistik der Sanitätsverhälstnisse der Mannschaft des k.u.k. Heeres*		*Sanitätsstatistischer Bericht des k.u.k. Heeres*
**Years of publication**	1870–1894		1895–1904		1905-–912
**Development**	Ministry of War				
**Basis**	Basic data				
**Frequency**	Annually				
**Publisher**	Der kaiserlich-königlichen Hof- und Staatsdruckerl				
**Pulmonary tuberculosis (data presentation)**	Morbidity and mortality in relative and absolute terms		Morbidity and mortality in relative and absolute terms		Since 1908, morbidity and mortality in relative terms.
**Other forms of tuberculosis** **(data presentation)**	Since 1884 morbidity and mortality in relative terms				
**Scrofulosis** **(data presentation)**	Morbidity and mortality in relative and absolute terms		Since 1894 included in the category “Other forms of tuberculosis”		-
**Comments**	In the footnotes as MSJ		In the footnotes as SSMH		In the footnotes as SBH

Basic data					
Type		Development	Frequency	Basis	Comments
Medical book (*Marodenbuch*)		Branch	Current	-	Closed at the end of the year
Soldiers’ disease protocol (*Manschaftskrankenprotokoll*)			Monthly	*Marodenbuch*	
Patient admission protocol (*Krankenzugangsprotokoll*)		Ward hospital	Current	-	
Patient report (*Truppenkrankenrapport*)		Branch	Monthly	*Manschafts-krankenprotokoll*	If there is only one unit stationed in the garrison, it acts as a garrison report
Garrison medical report (*Garnisonskrankenrapport*)		Garrison		*Truppenkranken-rapport*	-
Corps medical report (*Truppenkrankenrapport des Korps*)/		Body	Monthly	*Truppenkranken-rapport,* *Garnisons-krankenrapport*	Sent to the Ministry of War
Report of medical corps hospitals (*Spitalskrankenrapport des Korps*)		Corps hospital			

Source: *Vorschrift für die sanitätstatistischen Eingaben im k.u.k. Heere*, (Wien: Die kaiserlich-königliche Hof- und Staatsdruckerl, 1904), 2-5, 11-12, 16-19.

The effectiveness of the above system was dependent upon the administrative and documentary diligence at the lowest levels; the risk of errors in the various files being particularly possible, if not highly likely, due to staff shortages. For example, even though at the beginning of the 20th century, civilian doctors were allowed to provide medical care for ill soldiers, which also involved record keeping,^26^ they were so burdened with other duties, that they could not always exercise due diligence in maintaining military statistics. That said, determining the scale of errors in military statistics at the lowest levels is not possible due to the fact that no raw statistical data has survived in the records of the common Ministry of War.

## The Organisation of the Austro-Hungarian Armed Forces

The Austro-Hungarian armed forces had a complex structure (on a peacetime basis, they were divided into four parts under separate ministries). This article presents the sanitary situation of the joint army (*gemeinsame Wehrmacht* depended on the joint Ministry of War—*k.u.k. Kriegsministerium*) also referred to in the text as the Imperial and Royal Army.^27^ It was formed by means of universal conscription, which was introduced by a law of 1868, and applied to all men who had reached the age of 20. Although between 1868 and 1912 military service lasted three years in all branches of the army, it was shortened for the infantry to two years in 1912.^28^ During the period in question, between about 90,000 and 125,000 young men were drafted annually into the joint army, while at the same time a similar number of soldiers were discharged after completing their basic military service.^29^ This state of affairs amounts to a cyclical population flow between the armed forces and civilian society.

Enlisted men were placed in units formed on the basis of the territorial principle of enlistment. At the outbreak of the First World War, for example, the territory of Austria-Hungary was divided between 102 regimental districts (each comprising several counties) constituting the recruiting base for an infantry regiment. At least part of the regiment was stationed within its district. Troops of other branches of the army (e.g. cavalry or artillery) were formed on the basis of several regimental districts or an entire corps district.^30^ The latter, also called military territorial districts (*Militärterritorialbezirken*), were sixteen in number, each constituting a recruiting base for a single corps. On several occasions the size and location of the corps districts were changed respecting the border between Cisleithania and Transleithania.^31^

The general obligation to serve in the army, together with the territorial basis of unit organisation, meant that the population characteristics of individual regimental districts were strongly marked in the regiments. In addition, regiments were highly differentiated in terms of their social make up (e.g. some were made up of undernourished recruits from the Carpathian countryside, while others received urban residents). The above state of affairs determined the magnitude of the risk of recruits infected with tuberculosis being drafted into the army and the susceptibility of drafted reservists to infection.

The varying health status of recruits from different districts and the desire to keep the army in good shape forced the conscription committees to conduct physical examinations of recruits, as regulated by the regulations of 1869, 1883, 1889 and 1912.^32^ Based on the surveys, four categories of recruits’ fitness for military service were specified, namely: A—fully fit for military service (*tauglich*); B—also fit for military service despite being in poorer physical condition (*mindertauglich*); C—unfit for service with arms (*zum Waffendienste untauglich*); and D—unfit for military service (*untauglich*).^33^ In addition, the instructions contained a catalogue of medical conditions that constituted exemption from service, which also included tuberculosis, with its pulmonary form closing the way to A and B categories at least. The examinations aimed at revealing a conscript’s ill health were relatively straightforward. In the case of the thorax, they focused on its degree of normal shape, in addition to a series of measurements down to the observation of one’s freedom to breathe (in case of doubt, the examination was supplemented by auscultation or percussion). On the other hand, examinations of the skeletal system, also relevant in the context of tuberculosis, boiled down to observations of the recruit’s limbs and posture in movement.^34^ As these methods were far from perfect, they may have translated into low detection rates of activated but not yet fully developed TB. The above instructions were rarely amended, not even influenced by new medical discoveries (even the 1912 instruction did not mention the possibility of diagnostic use of the X-ray machine). Although this state of affairs speaks in favour of the relatively early establishment of the medical basis of military service, it also testifies to the considerable conservatism of Austria-Hungary’s imperial military authorities.

Military medical examinations were subject to superarbitration (this covered soldiers in active service, post-service conscripts and recruits in reserve), whose aim was to confirm unfitness for service on the grounds of impairments or persistent health problems. The arbitration committee determined the degree of unfitness and the amount of compensation (disability pay/pension) to which those subject to discharge were entitled. The regulations on arbitration were changed even less often than the instructions for conscription committees. The set of regulations on arbitration drafted in 1885 still remained in force in 1914.^35^

## Tuberculosis in Austro-Hungarian Society

The scale of the TB problem in Austria-Hungary was overwhelming. In Cisleithania alone between 1882 and 1913, the number of patients who completed hospital treatment due to multiform TB increased over 3.5 times, from just under 17,000 in 1882 to over 60,000 in 1912 (although the following year bought a decrease by 17%) – at the same time the Cisleithania population increased from approximately 22,000,000 to around 28,500,000. Simultaneously, the number of patients who died during treatment increased from approximately 8,000 to over 10,000; nonetheless, due to an increase in the number of hospital beds for TB patients the percentage of deaths decreased (from approximately 43% in 1886 to approximately 19.5% in 1912, with an increase in 1913 to 20.5%).^36^

In 1896-1913, in Austria-Hungary, TB-related death numbers were considerably higher than the number of patients under treatment, and fluctuated between approximately 150,000 and 187,000 cases per year, reaching its peak in 1905 (the population of Austria-Hungary in 1900 was about 45,500,000 people, and had increased ten years later to about 49,500,000 people). The disease took a higher toll in the Habsburg Crown Lands, the difference being between approximately 7,000 to nearly 20,000 cases per year. The proportion of tuberculosis-related deaths in the general mortality figures in both parts of the Dual Monarchy was, nevertheless, similar – in Cisleithania this fluctuated between 13 and 14%, and in Transleithania between 13 and 16%, which made TB the most lethal of infectious diseases afflicting Austro-Hungarian society.^37^

## Tuberculosis in the Austro-Hungarian Common Army

**Figure 1.**
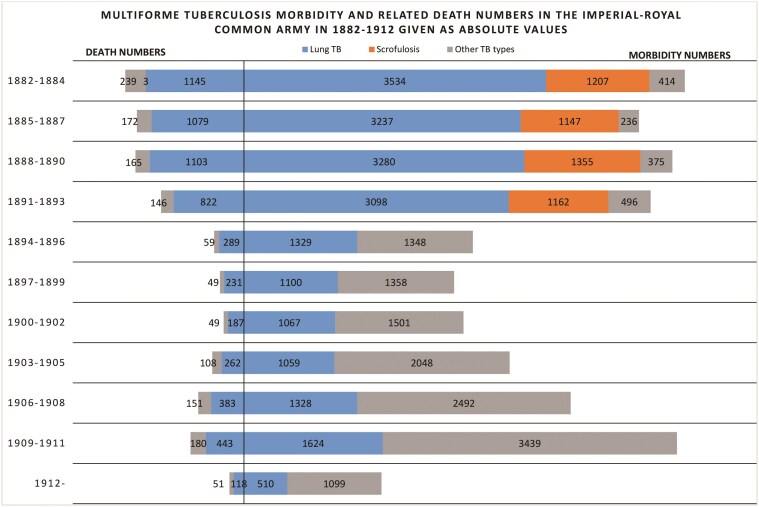
Source: MSJ 1880, 1881,1882, 344-345; MSJ 1883, 1884, 22-23, 194-195; MSJ 1885, 230-231; MSJ 1886, 206-207; MSJ 1887, 222-223; MSJ 1888, 212-213; MSJ 1889, 216-217; MSJ 1890, 220-221; MSJ 1891, 218-219; MSJ 1892, 226-227; MSJ 1893, 238-239; MSJ 1894, 274-275; SSMH 1895, 126-127; SSMH 1896 128-129; SSMH 1897, 132-133; SSMH 1898, 130-131; SSMH 1899, 126-127; SSMH 1900, 126-127; SSMH 1901, 120-121; SSMH 1902, 114-115; SSMH 1903, 110-111; SSMH 1904, 110-111; SBH 1905, 110-111; SBH 1906, 108-109; SBH 1907, 106-107; SBH 1908, 47 SBH 1909, 47; SBH 1910, 49; SBH 1911, 49; SBH 1912, 55; MSJ 1908, 143, 145; MSJ 1909, 143, 145; MSJ 1910, 143, 145; MSJ 1911, 143, 145-146; MSJ 157, 159-160.

Figure 1 illustrates the number of TB cases in the Common Army in 1882-1912. As many as 40,843 cases of multiform tuberculosis were detected, which gives a mean of 1,317 annually (lung TB was the most common among soldiers—51.8% of diagnosed cases). The lowest morbidity was noted in 1899, and the highest in 1883, fluctuating between 775 and 1,871 cases, respectively. The largest amount of cases was detected in 1901–1912, comprising 15,341 patients, i.e. as high as 37,5% positively diagnosed cases. In the previous decade, over 4,000 fewer cases had been detected, while between 1882 and 1890 there were 14,785 diagnosed cases. At the same time, the size of the army ranged from around 265,000 to 300,000 soldiers.

In the last decade before the First World War, the annual morbidity rates started to rise again. This was partly due to an increase in the annual recruitment quota and a change in the number of cases of each form of tuberculosis detected. In 1882–1894, lung tuberculosis dominated all the other forms; although in the last year of this period, this dominance was not so clear-cut (54 cases). In the following years, the dominance of other forms of TB became more pronounced.

First, this was caused by a reduction by approximately 40% of positive lung TB diagnosed cases in the Common Army (from 958 cases in 1,893 to 528 a year later). Most probably, the fall was related to the new health care instruction for military barracks issued in 1894 (see below). The introduction of the new regulations had the effect of improving the living conditions of recruits by reducing the development of disease in infected individuals. In addition, it reduced the risk of pulmonary tuberculosis infection in the army. Probably the second reason for the predominance of extrapulmonary forms of tuberculosis was the discovery of more cases of disease located outside the respiratory system, thanks to the development of diagnostics.

The army, thus, was not exempt from the broader death toll from tuberculosis. In 1882–1912, *Mycobacterium tuberculosis* caused 7,434 deaths (approximately 240 per year), meaning an 18% mortality. The highest number of deaths was noted in 1883—528—and the lowest in 1900, at a mere 71. Death rates peaked in the 1880s and the beginning of the 1890s with their curve showing a downward trend up until 1904. The most probable cause was the above-mentioned improvement in living conditions and effective superarbitration. As a result of diagnostic advancement, the latter facilitated more frequent incidences of discharge from service prior to the terminal stage TB, thus eradicating a potential epidemiological threat in the army.

Lung TB was the most effective killer and caused 6,062 deaths (approximately 81,5%) and prevailed even after a significant reduction in detected case numbers. However, scrofulosis-related death rates were at a minimum trace level, with just 3 cases.

Between 1882 and 1912, TB mortality in the army was lower than in civilian hospitals, where mortality rates oscillated between approximately 19.5% to over 43% of TB patients.^38^ In the army, however, the percentage did not exceed 30%, and, after 1893, did not rise above 20%, which constitutes a difference from 12 to nearly 25 percentage points. This situation was caused, most of all, by the above-mentioned service discharge and sending soldiers on leave in the terminal TB stages, as well as improved military hospital conditions. Between 1904 and 1905, however, there was an increase in TB mortality in the army (more than twice that of pulmonary TB and five times that of other forms of TB). This development should most likely be linked to improvements in diagnosis, which made it possible to attribute deaths of previously undetermined cause; or those erroneously attributed to other diseases to tuberculosis.

Approximately 65–80 per cent of TB cases were noted in Cisleithania. The disproportion between the two parts of the Dual Monarchy was also noticeable in the death rates.^39^ These variances may have stemmed from inaccuracies in the statistics collected in Hungary; or conversely, may present actual differences in disease incidence.

The proportion of TB patients in the general army population in 1882–1912 is shown in Supplementary Table S1. In that time, TB patient numbers per every thousand soldiers fluctuated between 2.5 in 1901 and 7 in 1883, the median being 4.3, the arithmetic mean 4.37, and the mode 2.8 and 5.8.

In 1882–1912, the highest percentage of TB patients concerned medical units, rolling stock divisions and artillery technical units. Their morbidity rates not infrequently exceeded the median (70% of cases for medical and rolling stock units, 50% for artillery technical formations), sometimes exceeding it twofold. This was a result of the regulations on medical tests for conscripts issued in 1889, which classified many ailments (including TB); not as a premise for service incapacity, but as a counter-indication to weapon-bearing service.^40^ Therefore, it was because men of a weaker physique were enlisted into military medical units and rolling stock service that this translated into morbidity within these formations. Both services displayed another risk factor, that is frequent contact with human and animal pathogens, which could affect one’s immunity.

High morbidity in the artillery technical divisions was most probably caused by the army’s attempt to make use of soldiers’ technical capabilities for as long as possible. This resulted in postponing leave time or discharge. This approach contributed, however, to the spread of the disease.

Between 1882 and 1912, the mortality rate did not exceed 0.2, and did not fall below 0.024 percentage points, with a median of 0.058 per cent, an average of approximately 0.083 percent and a mode of 0.15 per cent.^41^

## Tuberculosis Incidence in the Civilian Population and the Imperial and Royal Army

Although the structure of civilian statistics makes it impossible to compare morbidity rates in the army and the wider civilian population, it is possible to compare mortality rates. Military service discharge for those most stricken resulted in this factor in the Common Army in 1900 being over 13.5 times lower than in the civilian population of the Dual Monarchy (excluding Bosnia and Herzegovina); however, in 1910, it was only 4.75 times lower. The reduction in divergence came, above all, from the noticeable increase in morbidity and mortality due to multiform tuberculosis in the army after 1905, and an increase of the population in Austria-Hungary during the 10 year period by about four million (a divisor increase).^42^

A potential correlation for TB incidence in the Cisleithanian population and the Common Army corps can be illustrated by comparing civilian mortality rates with morbidity and mortality among soldiers. The source database allows this only for the period of 1896-1907. Based on the divergence of civilian mortality rates (as per data from 1910), the comparison covers regiment districts number: 4 (Vienna), 11 (Písek; covered districts: Pisek, Blatná, Klatovy, Sušice, Strakonice) and 20 (Nowy Sącz; covered districts: Nowy Sącz, Nowy Targ, Limanowa, Grybów, Gorlice). The selection of exemplary regimental districts serves the purpose of presenting the threat of tuberculosis and the possible parallel nature in this field between the civil society and the army in areas presenting different degrees of social and economic development. These districts varied in terms of population density, the predominant type of economy and the type of housing. The area of the 4th Infantry Regiment district included the capital of the Dual Monarchy, the largest urban centre of the Habsburg state (2,031,498 inhabitants in 1910), which, together with the neighbouring districts, was an important industrial centre. In contrast, the districts of Písek (332,240 inhabitants in 1910) and Nowy Sącz (428,739 inhabitants in 1910) were characterised by their rural nature, with the countryside of district 20 infantry regiment, as in other areas of Galicia, often overcrowded and dominated by fragmented land ownership.^43^

At the beginning of the twentieth century, the percentages of men conscripted in the 4th, 11th and 20th regimental districts varied and differed over time. In the Vienna regiment, they ranged from 11 per cent in 1906 to 26% in 1900, and in the Písek Regiment from 15.7% in 1908 to 29.2 in 1901. Against this background, the Nowy Sącz Regiment clearly stood out, which in the period 1900–1910 never once conscripted more than 17% of the men subject to compulsory military service; at times, even falling below 10 per cent.^44^ These differences were due to several factors, such as the size of the conscript cohort for that year or the immediate demand of infantry regiments for recruits. It seems, however, that the most significant of these was the general health of the men standing before the recruiting committees. This would explain the consistently low percentage of able-bodied recruits in the Nowy Sącz regimental district as Galicia stood out for its low standard of living compared to other parts of the Cisleithanian region.

It should be emphasised that interactions between the army and the civilian population, not only in the above-mentioned districts but in the whole of Austria-Hungary, were not limited to the recruitment of recruits. The interaction between civilians and soldiers in the garrison towns of the Dual Monarchy can be divided into official and unofficial. The former category included the participation of the armed forces in state ceremonies, their assumption of the functions of fire brigades and law enforcement, and the military’s renting of accommodation from private owners. The latter aspect mainly concerned officers and, in relation to soldiers of lower rank, disappeared with the expansion of the barracks network, although the army reverted to taking out leases on private buildings during periods of conducting manoeuvres when the number of soldiers in particular areas of the state increased. In the context of the epidemiological threat, the army’s relations with the civilian population on an unofficial level should be considered more important. This included the participation of soldiers, especially officers, in the social life of garrison towns and the various forms of leisure activities of soldiers related to catering or establishments of ill-repute.^45^

The presence of TB in the Vienna, Písek and Nowy Sącz regimental districts was more than evident. In 1896–1913, TB accounted for the following mortality rates among men in particular regimental districts: infantry regiment 4—approx. 8–9 per cent, infantry regiments 11 and 20—approx. 1 per cent each^46^. Yet, among men of conscription age (20-30 years of age), this caused approximately 60 per cent deaths in the Vienna district and the Czech lands (although the Czech lands data presented higher figures, data for particular counties is missing). In Galicia, the TB-related death rates among young men did not exceed 50% due to a higher number of deaths caused by other factors in that age group in this region. It should be noted that among male TB patients in Cisleithania, those aged 20–30 years were the most numerous group of victims (each year there were approximately 7,500–8,500 individuals representing 17–19 per cent of male victims of Koch’s bacillus)^47^.

**Figure 2.**
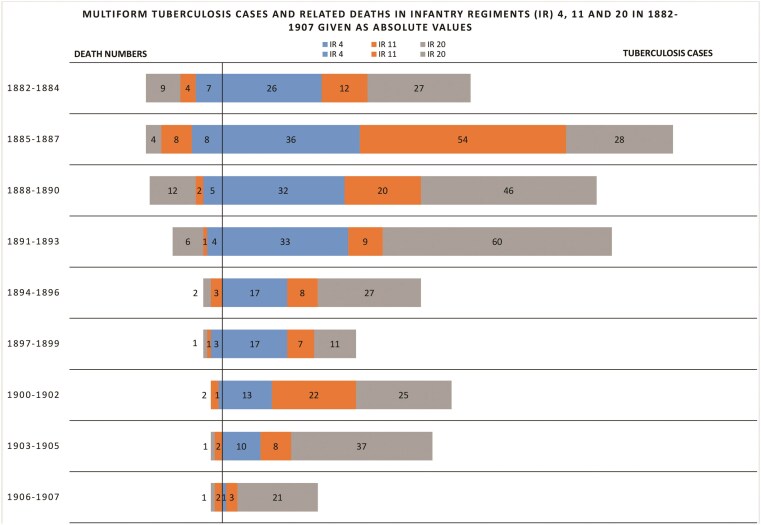
Source: MSJ 1880, 1881,1882, 348, 352, 356-357, 365; MSJ 1883, 1884, 26, 30, 34, 39,198, 202, 206, 211; MSJ, 1885, 234; 238, 243; 251; MSJ 1886, 210, 214, 219, 227; MSJ, 1887, 226, 230, 235, 243; MSJ 1888, 216, 220, 225,233; MSJ 1889, 220,224,225,229,233; MSJ 1890, 224, 228, 229, 233, 237; MSJ 1891, 226, 227, 230,231,235; MSJ 1892, 230, 234 238, 239, 243; MSJ 1893, 242, 246, 247, 250, 255; MSJ 1894, 304-306; SSMH 1895, 156-157; SSMH 1896, 158-159; SSMH 1897, 162-163; SSMH 1898, 160-161; SSMH 1899, 156-157; SSMH 1900, 156-157; SSMH 1901, 150-151; SSMH 1902, 144-145; SSMH 1903, 140-141; SSMH 1904, 140-141; SBH 1905, 140-141; SBH 1906, 138-139; SBH 1907, 136-137.

Figure 2 presents the number of cases and deaths caused by multiform tuberculosis in infantry regiments 4, 11 and 20 in 1896–1907. In those periods, the summary curve representing regimental morbidity displayed trends convergent with those in the entire Common Army. Moreover, the number of TB-related deaths within regiments was not high. In order to verify the metaphor of the conscription army as a reflection of the condition of the health of society as a whole, it is necessary to run a separate data analysis for particular regiments.

Between 1882 and 1907, a small percentage of the soldiers of the 4th Infantry Regiment suffered from tuberculosis (only in 1886 and 1893 did the number of patients significantly exceed 10–12 cases^48^). Thus, the health status of the soldiers of the Viennese Regiment does not reflect the difficult sanitary situation of the city, which saw the largest TB outbreak in the Dual Monarchy. The apparent discrepancy between the sanitary condition of the regiment and the mobilisation facilities was due to several factors. The decrease in the morbidity of soldiers, as discussed further on in, was influenced by regular high-calorie meals (not always available to the poorer sections of the urban population) and housing conditions (living in uncrowded and often ventilated rooms). The thoroughness of the examination of recruits was probably also not without significance—in a tuberculosis-ridden recruiting district such as Vienna, military doctors probably paid particular attention to the symptoms of the disease by resorting more often to palpation and auscultation as diagnostic methods.

Among the soldiers of regiment 11, conscripted in 1882–1907 from an area with relatively low TB mortality rates, tuberculosis morbidity fluctuated between high figures to nearly single digits. Equally, at the end of the period under research, the situation in the regiment constitutes a true reflection of the condition of health in the population of the area, a phenomenon which had not happened before. The high morbidity within the infantry regiment—11 soldiers at the end of the nineteenth century—begs the question as to the accuracy of the civilian statistics. Thus, it is possible that the actual state of health of the population of the infantry regiment 11 area could only be reflected in the military statistics. However, verification of this proposition requires further research.

In infantry regiment 20, a clear discrepancy emerged between a low TB contribution to the number of deaths among the area’s population (including young men) and the number of soldiers suffering from TB. Except for 1896–1899, (only a few cases per year) a relatively high number of multiform TB patients was noted in infantry regiment 20. The difference came as a result of special circumstances of the Carpathian lowlands, which were plagued by TB forms different than scrofulosis or lung TB, and which made diagnosis difficult. Access to doctors was difficult in the area, which also contributed to the overall situation; and so, enlistment often represented the best possibility of receiving a correct diagnosis.

## Tuberculosis Treatment and Prophylaxis in the Imperial and Royal Common Army

In the Common Ministry of War, Division 14 (*Abteilung 14*) was in charge of health and hygiene issues under the command of the Chief of Military-Medical Officer Corp (*Chef des militärärztliches Offizierskorps*), and a consulting function belonged to the 10-man-strong Military Sanitation Commission (*Militärsanitätskomitee*). Care for the sick was provided within medical wards (*Krankenabteilungen*) at 27 garrison hospitals (*Garnisonsspitaler*), some of which also maintained branches. Military treatment facilities also included division hospitals (*Truppenspitäler*) and homes for the sick (*Marodenhäuser*). The former were organised within garrisons of more than 500 soldiers and did not constitute garrison hospital headquarters. The latter, formed in garrisons of 300–500 soldiers, catered for less serious cases, and looked after patients who were not fit to be transported.^49^

The treatment facilities staff came from the Military-Medical Officer Corp, to which volunteer-doctors belonged (having accepted a government grant to complete study programmes or later claimed the reimbursement of the study expenses), and who were obliged to perform seven years of service. As the army, however, struggled to fill medical vacancies, civilian doctors were sometimes called upon. Lower ranked staff had a core of adequately trained non-commissioned officers and line troops.^50^

It should be emphasised that, at the turn of the twentieth century, the armed forces were among the few entities that could provide their members with access to permanent and free medical care. Thus, within the ranks of the military, it was easier, if not to attempt to treat tuberculosis, then at least to diagnose it. Unsurprisingly, the threat carried by Koch’s bacillus forced the military medical services to attempt to treat TB and limit its spread. A wide range of measures from pharmaceuticals to climatic therapies to prophylaxis would be employed to serve this purpose.

Also at the turn of the twentieth century, no effective and human-safe anti-tuberculosis drugs were known (streptomycin was not discovered until the 1940s^51^). Some of the medicines used were aimed at treating the symptoms of tuberculosis. This was the case with laudanum (according to the Austro-Hungarian military pharmacopoeia, a mixture of wine spirit, distilled water and opium corresponding in properties to approximately 1 g of morphine^52^), which was supposed to relieve the pain accompanying tuberculosis. The remedy was also used as an analgesic in the treatment of other conditions.

Causally, attempts were made to treat pulmonary tuberculosis with inhalations designed to restore the lungs’ normal function. Inhalation mixtures varied widely, with ingredients including light gas, hydrogen sulphide, creosote iodine, turpentine and phenol. Sometimes it was not so much the composition of the mixture that was important, but its temperature, which had to do with the discovery of the sensitivity of pathogenic microorganisms to heat.^53^ Treatment with the above agents, however, led to a poisoning of the patient and a further weakening of the body.

Less dangerous was the use of calcium in therapy (this was also part of the climatic treatment). This was used to strengthen the shells surrounding tuberculosis foci in the lungs^54^.

Gold was also used in the treatment of tuberculosis. Its effectiveness, in vitro, was confirmed by Robert Koch’s experiments at the end of the 19th century (gold cyanide reducing bacterial growth). On a wider scale, gold preparations appeared in treatment shortly before the outbreak of the First World War.^55^

Although there is no indication that gold therapy was used in the Imperial Army, the preparation of other anti-tuberculosis drugs was possible based on the resources of military pharmacies.^56^ Various forms of drug treatment were given to at least some of those soldiers who ended up in military hospitals (in 1885, this comprised about 60% of diagnosed cases of pulmonary tuberculosis^57^). Unfortunately, the state of preservation of the source base does not make it possible to determine which of the remedies was most readily applied.

Attempts were also made to treat pulmonary and arthritic tuberculosis through climatic therapies by placing the patient in a spa with conditions that made it difficult for mycobacteria to grow. Such facilities usually specialised in a specific type of therapy and the treatments included: relaxation in the open air, salt water baths, proper diet (inclusion of mineral waters in meals) or heliotherapy (consisting of sunbathing and aimed at tuberculosis of the skin and joints). The aim of the above treatments was to strengthen the patient’s immune system. The most important Austro-Hungarian tuberculosis sanatoria were located in Baden, Baden Hall, Roznau, Grado, San Pelagio bei Rovigno or Sulzbach-Ischl. In Austria-Hungary, sick soldiers were referred to civilian institutions. The length of treatment determined that soldiers undergoing sanatorium treatment were previously dismissed from service or on leave^58^

Surgical operations were also intended to improve the condition of tuberculosis patients. The manner of construction of military statistics makes it impossible to determine how many Austro-Hungarian soldiers underwent surgical operations due to tuberculosis. Before 1884, and from 1905 onwards, the yearbooks do not record the reasons for the operations; and even before that, these data were not always published.

Between 1884 and 1912, the most common operations performed in the Austro-Hungarian army were related to the treatment of pulmonary tuberculosis. Although for reasons mentioned above it is impossible to determine their exact number, a significant proportion of the 2,339 thoracic operations performed between 1884 and 1912 were related to this disease. Soldiers suffering from illness underwent thoracotomy (opening of the chest), thoracocentesis (puncture of the pleural cavity, in the case of tuberculosis to evacuate an abscess or to induce emphysema) and rib resection.^59^ In contrast, the lung resection procedure, characteristic of late nineteeth- and early twentieth-century TB treatment, was not performed in the joint army.^60^ This was because such surgery was performed on patients with advanced tuberculosis, who were most often discharged from the army.

Other forms of tuberculosis were also treated surgically. It is worth noting that operations related to bone tuberculosis were not particularly numerous. Between 1884 and 1904, only 41 amputations were carried out citing this as a reason, representing less than 1 per cent of all operations carried out in the army. Other operations associated with this form of the disease included resection and bone lancing or surgical intervention in the joint region. In addition, in 1900, military surgeons performed one spinal trepanation for tuberculous reasons.^61^

According to military documentation, at least some of the above surgeries ended with the patient’s recovery, which was equivalent to a return to service.^62^ This, however, was an over-interpretation. While facilitating patients’ functioning, procedures of this type eradicated the most serious inflammatory foci, it did not solve the core issue, as *Mycobacteria tuberculosis* still remained in the patient’s body.

This inability to cure TB forced one to search for other methods in order to deal with the infection. Restricting disease foci in the army was facilitated by a series of procedures, both intentional, and those connected with other than sanitary areas of armed forces life.

The most effective solution to TB in the army would be total removal of TB patients, especially those still spreading the disease, from the ranks. The Habsburg armed forces’ stance in the regard seems ambiguous. The conscription examination regulations of 1889 did not class TB-affected people to be incapable to serve in the army, which, at the same time, only closed off the possibility of weapon-bearing service (the regulations contained the phrase *die Tauglihkeit zum Waffendienste aufheben*, which means revoking the right to weapon-bearing service), and thus forced allocation to sanitation, transportation or army base workshop services. The stipulation concerned also lung TB, which was a significant epidemiological threat. Only the regulations of 1912 deemed TB patients as totally incapable of military service and extended the catalogue of forms of TB that were grounds for discharge from service.^63^ This only happened 30 years after Koch’s initial discovery.

The above-mentioned change would entail a slow, yet clear evolution of the army’s views on the mere presence of TB in the armed forces and the ways of limiting its impact. However, this image is obscured by the superarbitration regulations of 1885, which co-exist with the conscription examinations regulations of 1889. At the time, lung TB and scrofulosis were considered sufficient for discharge from service (most certainly, later stages of TB were interpreted this way). The same regulations failed to mention, however, other types of TB.^64^ Parallel application of both sets of regulations in the army must, therefore, have seemed inconsequential.

What is also worth considering is the practical application of the superarbitration stipulations for TB purposes. The structure of the military statistics allows a detailed study of the mutual correlation between multiform TB patient numbers and the number of Austro-Hungarian soldier deaths, those sent away on leave, or discharged from service due to TB in 1882–1894. During this time, from 36.5 to nearly 60% of TB-affected individuals remained in the army (1894). In terms of absolute values, most lung TB patients remained in service, whereas in percentage terms, until 1887, the primacy belonged to other types than lung TB and scrofulosis (also coming first in this category in 1888). This was, most certainly, due to the difficulty in diagnosing other TB types.^65^

It is challenging to indicate the causes as to why the Austro-Hungarian military authorities kept TB patients in service. Perhaps the army attempted to keep in service those soldiers who were specialists in certain areas in order to avail of their expertise (a similar phenomenon may be observed in the professional armies of the countries that were defeated in the Great War; in their cases, this was a necessity^66^). This reason, however, does not seem very probable, as it would have meant consent to frequent and nearly constant mycobacterial disease incidence in certain military communities. In the most serious cases, soldiers who continued to spread TB were discharged, as they posed the gravest epidemiological threat. Less advanced TB patients were kept on, without regard to the fact that should living conditions deteriorate, they could become disease incubators. That said, the above policies of the Habsburg military authorities were somewhat associated with the recognition of TB as a chronic disease.^67^

The effectiveness of removing tuberculosis patients from the army was diminished by diagnostic problems because, as mentioned above, Koch’s bacillus did not always immediately reveal its presence in the body, and its detection required specialised methods. At the dawn of the 1800s, the examination to establish tuberculosis was still based on palpation and auscultation of the patient’s chest.^68^ Observation of his temperature and body weight were also helpful in the diagnosis. With the slow introduction of more modern methods (as discussed further below), the set of steps above were the basic tools for diagnosing tuberculosis in the Imperial Royal Armed Forces until the outbreak of the First World War.

The use of tuberculin (a protein extracted from cultures of the tubercle bacillus), isolated by Robert Koch in 1890 for diagnostic purposes, proved to be an important change in the detection of tuberculosis; and the Austro-Hungarian military experimented with the use of tuberculin for TB diagnosis (it should be emphasised that this research was conducted independently of parallel civilian research). Two regiments were included in the experiment: the 1st Bosnian-Herzegovinian Regiment (in which the trial was carried out twice with a one-year interval) and the 60th Infantry Regiment (sampled once). The trial consisted of injecting soldiers with a significant dose of tuberculin (1 mg). After a period of time, a second dose of 2 mg was administered (soldiers with a negative reaction received a three-milligram dose). For about two days after the injection, the body’s reaction (mainly temperature changes) was observed. The Bosnian Regiment also monitored changes in the subject’s weight between trials. The reaction was to provide information on a possible primary infection with Koch’s mycobacterium, which agreed with Clemens von Pirque’s view of the effect of tuberculin. The study revealed the scale of the TB problem in the Austro-Hungarian armed forces. In 1902, 108 soldiers in the 60th Infantry Regiment tested positive, while according to the statistical yearbooks, nine soldiers in the regiment were suffering from tuberculosis. This would prove that the army was ignoring the problem of inactive TB. However, the burden of this allegation diminishes in light of the fact that a soldier in whom the disease had not developed did not pose an epidemiological threat. The effectiveness of the first tuberculin tests also remains debatable, as it used unpurified tuberculin: it appears that this may have led to false-positive results. The occurrence of a similar problem seems to be confirmed by the contents of a letter addressed on 16 January 1903 to the Ministry of War of the command of the Imperial and Royal II Corps, which emphasised that there had not been a single case of spontaneous cure of latent tuberculosis that year. Such a record suggests that, at times, test results indicated the occurrence of a similar situation that is medically impossible.^69^

Beside tuberculin, another modern TB diagnostic method was the X-ray. However, even on the eve of the outbreak of the First World War, not all of the 27 Austro-Hungarian garrison hospitals had equipment necessary for this procedure. This situation did not only characterise the Dual Monarchy. Indeed, in Germany, despite its higher level of development, X-rays as part of TB diagnostics only came into common use in the army after the First World War.^70^

Tuberculosis prevention in the Austro-Hungarian army was not limited to the dismissal from the ranks of patients in the most serious condition. The level of morbidity was also influenced by the living conditions of the soldiers of the joint army. This aspect of the functioning of the armed forces, however, did not undergo modernisation as quickly as the model for recruiting soldiers. Firstly, as late as the mid-1870s, some 34,000 Habsburg soldiers (almost 16% of the ground forces) still lived outside barracks. Moreover, most of the barracks were not buildings designed for this purpose, but castles, secularised monasteries or other civilian buildings. Secondly, even after the military authorities recognised the problem of a shortage of quarters, barracks construction was dominated by defensive buildings, characterised by cramped internal courtyards and limited access to fresh air; and prevailing cold and damp. Combined with omnipresent dampness, this did not create the conditions to reduce the incidence of tuberculosis. The situation changed in the 1870s with the introduction of new barrack designs based on the British experience of the Crimean War. Barracks with a pavilion layout provided, among other things, a constant supply of fresh air. Barrack camps, erected from the second half of the 1880s onwards as a result of the rapid growth of the armed forces combined with budgetary shortfalls, had similar features.^71^

For the prevention of the spread of TB, no less important than the nature and layout of the military accommodation was its maintenance. The above-mentioned dramatic fall in lung TB morbidity between 1893 and 1894 stemmed from the implementation of the new instructions on hygiene maintenance in military boarding houses. Although the previous instruction of 1881 had already pointed to certain behaviours which could help limit the spread of TB; it was only the regulations of 1894 that placed a particular emphasis on the issue. Admittedly, the previous stipulations had mentioned the need of long-duration room airing and for spittoons in the barracks. At the same time, clean floors were deemed unnecessary (they were merely sprinkled with sand) as long as soldiers removed and left their shoes by the door upon entry. The new instructions strictly ordered soldiers to air rooms twice a day (in the morning and after dinner). The same frequency was to be applied to dusting and cobweb removal, cleaning floors, windows, doors, tables, benches or beds, and changing sand in spittoons. These activities massively contributed to a shorter potential exposure of soldiers to Koch’s bacillus within garrison interior spaces. It should be noted that these TB instructions were identical with those aimed at civilians.^72^ The implementation of the above recommendations of the regulations was guaranteed by the regular inspection of the cleanliness of the soldiers’ chambers by those non-commissioned officers responsible for the training and upbringing of recruits, which extending to rigorous cleaning duties. The actions of the NCOs were in turn monitored by the officers holding command of the garrison.

Another set of instructions from 1909 included special chapters devoted to counteracting particular infectious diseases, including TB.^73^ This act can be deemed proof of a gradual evolution in the attitude towards the issue and its potential solution within the Imperial and Royal Armed Forces.

In addition to the construction of barracks and the introduction of orderly regulations, another factor which, if not limiting the spread of tuberculosis in the army, at least delayed its activation was the regular nutrition of soldiers. At the dawn of the twentieth century, an Austro-Hungarian soldier daily consumed 400 g of beef, 700 g of bread or 400 g of rusks, 140 g of rice, groats or legumes supplemented with coffee concentrate. The daily ration of liquids was 0.5 litres of wine and up to 4 litres of water. The regulations allowed a wide range of substitutes for all the products making up the ration. Its composition was based on the results of nutrition research undertaken at the time, which found that a soldier should receive about 3,500 calories a day from about 120 to 130 g of protein, 90–100 g of fat and 500 g of carbohydrates. It should be stressed that the military ration often provided a greater energy load than the daily food of the civilian inhabitants of the Dual Monarchy. This was the case, for example, in the case of Galicia, whose rural population consumed around 2,000 calories per day, derived mainly from vegetable products.^74^ In addition to the fact that, in terms of composition and caloric content, military meals may have been attractive to recruits from the poorer provinces of the Dual Monarchy (above all Galicia, Bukovina and Upper Hungary), their regularity and abundance must have bolstered a soldier’s chances of not contracting tuberculosis.

## Conclusion

As one of the most widespread social ailments of the nineteenth century and the first half of the twentieth century, tuberculosis did not leave the Imperial and Royal Common Army unscathed. In period 1882–1914, the morbidity curve among Habsburg soldiers for diseases induced by Koch’s bacillus and bovine Mycobacteria fluctuated from high figures to low; before rising again towards the end of the period under investigation. The mortality curve behaved in a similar fashion. It should be noted that a temporal correlation between the most drastic changes to the extent of TB in the Imperial and Royal army and discoveries in microbiology or medicine remains impossible to prove.

The example cited above of three infantry regiments and districts (approximately 3 per cent of all regiment districts) from Cisleithania proves frequent and significant divergences between the extent of TB among residents of a given area and a military unit formed from among them. This situation is connected with examinations performed by conscription committees, wherein if recruit health checks were done diligently, the numbers affected by diagnosable TB entering the ranks was low. Moreover, any statement that TB morbidity and mortality were high in a regiment district, often led to an increase in detailed examinations carried out by the committees. In such cases, any trace of the state of health of wider society is to be found most of all in the documents of conscription committees. And so, when searching for related information in military statistics, we must proceed with caution. The actual reflection of the scale (active and latent) of TB incidence in society derives from tuberculin trial results performed within regiments. The preserved data concerns, however, only single regimental districts and is limited to relatively short periods of time (a year or two).

What emerges from the source material is an ambiguous picture of the attitude of the Austro-Hungarian military authorities to the problem of tuberculosis in the ranks of the army between 1882 and 1914. The conscript examination instructions in force for most of the period in question (the ability of TB patients to serve without weapons) and certain provisions of the superarbitration regulations (the possibility to leave TB patients to undertake treatment instead of definitively dismissing them from service), together with the long-standing practice of leaving a significant number of soldiers sick with TB in the ranks, suggest at the very least a downplaying of the danger posed by Koch’s mycobacterium. After all, it was not until 1912 that it was decided to completely remove TB patients from military service. On the other hand, however, in the last decades of the nineteenth century and at the beginning of the twentieth century, the Imperial Royal Armed Forces carried out anti-TB activities analogous to those of civilian institutions. These actions manifested both in the introduction of state-of-the-art preventive measures (e.g. regular airing and vacuuming of living quarters) and in research into possible diagnoses of the disease. It should be emphasised that the armed forces’ activities in the latter sphere, such as experiments with tuberculin, took place in parallel and independently of similar civilian initiatives. At the same time, observation, and the auscultation of recruits and soldiers suspected of having the disease remained the basic tools for diagnosing tuberculosis. Although advances in civilian and military research on tuberculosis would probably have led to the implementation of increasingly effective anti-tuberculosis provisions in the imperial armed forces, this process was halted by the outbreak of the Great War.

## Supplementary Material

Supplementary material mentioned in the text is available at www.shm.oxfordjournals.org.

hkae095_suppl_Supplementary_Tables_S1

## Footnotes

1 K. Waddington, *The Bovine Scourge*, 53–54; W. Brühl, R. Brzozowski, eds, *Vademecum lekarza ogólnego* (Warszawa: Państwowy Zakład Wydawnictw Lekarskich, 1990), 129; J. Furin, H. Cox, M. Pai, ‘Tuberculosis’, *Lancet,*2019, 393, 1642–1656.

2
*Sanitätbericht über das Reichsheer für das Jahr 1932 (1. Januar bis 31 Dezember)*, (Berlin: E.S. Mittler & Sohn, 1934), 81; MSJ 1885, 391; SSRKLJ 1882, 24.

3 Austria-Hungary was divided into the Habsburg Crown Lands (Cislithuania, which included Upper and Lower Austria, Styria, Carinthia, Salzburg, Tyrol, Vorarlberg, the Austrian Coast, Dalmatia, Bohemia, Moravia, Austrian Silesia, Galicia and Lodomeria, and Bukovina) and the Kingdom of Hungary (Translithuania), with the Litava River forming the border between the two.

4 R.Y. Keers*, Pulmonary Tuberculosis. A Journey down the Centuries* (London: Bailliére Tindal, 1978).

5 T. Dormandy, *The White Death. A History of Tuberculosis* (London, Rio Grande: Hambledon Press, 1999).

6 F. Ryan, *The Forgotten Plague. How the Battle against Tuberculosis Was Won—and Lost* (Boston, Toronto, London: Little, Brown and Company, 1992); *Tuberculosis The Greatest Story Never Told. The Human Story of the Search for the Curo for Tuberculosis and the New Global Threat* (Bromsgrove: Swift Publishers, 1992).

7 F. Condrau, M. Worboys, *Tuberculosis Then and Now. Perspectives on the History of an Infectious Disease* (Montreal & kingstin, London, Ithaca: McGill-Queen’s University Press, 2010).

8 F.B. Smith, *The Retreat of Tuberculosis 1850–1950* (London, New York, Sydney: Croom Helm, 1988).

9 T. M. Daniel, *Captain of Death. The Story of Tuberculosis* (Rochester: BOYE6, 1997).

10 K. Waddington, *The Bovine Scourge Meat, Tuberculosis and Public Health, 1850–1914* (Woodbridge: The Boydell Press, 2006).

11 C. Gradmann, ‘Robert Koch and the white death: from tuberculosis to tuberculin’, *Microbes Infect*., 2006, 8, 294–301.

12 D.S. Burke, ‘Of Postulates and Peccadilloes: Robert Koch and Vaccine (tuberculin) Therapy for Tuberculosis’, *Vaccine*, 1993, 11, 795-804.

13 J. A. Odell, ‘The History of Surgery for Pulmonary Tuberculosis’, *Thoracic Surgery Clinics*, 2012, 22, 257–69.

14 B. Biwald, *Von Helden und Krüppeln. Das österreichisch-ungarische Militärsanitätwessen und dessen Auswirkungen auf die Gesellschaft im Ersten Weltkrieg*, (unpublished PhD thesis, Vienna University, 2000).

15 J. D. Mancuso, ‘Tuberculosis Screening and Control in the US Military in War and Peace’, *The American Journal of Public Health*, 2017, 107, 60–67.

16 E. Dietrich-Daum, *Die ‘Wiener Krankheit’. Eine Sozialgeschichte der Tuberkulose in Österreich* (Wien: Verlag für Geschichte und Politik, 2007).

17 M. Baczkowski, *Pod czarno-żółtymi sztandarami. Galicja i jej mieszkańcy wobec austrowęgierskich struktur militarnych 1868–1914* (Kraków: Towarzystwo wydawnicze Historia Iagiellonica, 2003); ‘Armia jako czynnik modernizacji Galicji w dobie konstytucyjnej (1867–1914)’, in P. Franaszek, A. Nieczuchrin (*Problemy cywilizacyjnego rozwoju Białorusi, Polski, Rosji i Ukrainy od końca XVIII do XXI wieku*, (Kraków: Wydawnictwo Uniwersytetu Jagiellońskiego, 2007), 205–15; ‘Znaczenie armii austriackiej dla rozwoju cywilizacyjnego Galicji (1848–1918)’, *Kwartalnik Historyczny*, 2009, CXVI, 71–90; ‘Wojsko austriackie (austro-węgierskie) a miasta Galicji (1772–1918)’, in M. Baczkowski and T. Kargol*, Galicja. Studia z dziejów społeczno-gospodarczych*, eds. M. Baczkowski and T. Kargol, Kraków: Towarzystwo wydawnicze „Historia Iagiellonica”, 2017), 13–28.

18 J. Ch. Allmayer-Beck, “Die bewaffnete Macht in Staat und Gesellschaft“, in A. Wandruszka, P. Urbanitsch eds, *Die Habsburgermonarchie 1848-1918.* Band V: *Die bewaffnete Macht*, (Wien: Verlag der Österreichischen Akademie der Wissenschaften, 1987).

19 S. G. Higgens, *How was Richard Haldane able to reform the british army? An historical assessment using a contemporary change management model,* (unpublished PhD thesis, University of Birmingham, 2010).

20 E. M. Spiers, *The Late Victorian Army, 1868–1902* (Menchaster: Manchester University Press, 1992).

21 A. Mercer, *Infections, Chronic Disease, and the Epidemiological Transition. A New Perspective*, (Rochester: University of Rochester Press, 2014).

22 OeStA AVA Inneres MdI SA A 2477.

23
*Statistik des Sanitätswesens der im Reichsrathe vertretenen Königreiche und Länder für das Jahr 1884*, (Wien: Die kaiserlich-königliche Hof- und Staatsdruckerei, 1887) 62, 64—further in footnotes as SSRKLJ; SSRKLJ *1907, 1908, 1909, 1910*, 383; SSRKLJ *1896*, 2, 156-157; Magyar statisztikai évkönyv 1898. Ú.F. 6, (Budapest: Athenaeum, 1899), 37—further in footnotes as MSÉ.

24
*Ergebnisse der mit der Volkszählung vom 31. December 1880 verbundenen Zählung der häuslichen Nutzthiere in den im Reichsrathe vertretenen Königreichen und Ländern*, (Wien: Die kaiserlich-königliche Hof- und Staatsdruckerei 1882); *Die Ergebnisse der Volkszählung vom 31 December 1890 in den Reichsrathe vertretenen Königreiche und Länder* (Wien Die kaiserlich-königliche Hof- und Staatsdruckerei, 1892); *Die summarischen Ergebnisse der Volkszählung vom 31 December 1900 in den Reichsrathe vertretenen Königreiche und Länder* (Wien: Die kaiserlich-königliche Hof- und Staatsdruckerei, 1902); *Die Ergebnisse der Volkszählung vom 31 December 1910 in den Reichsrathe vertretenen Königreiche und Länder* (Wien: Die kaiserlich-königliche Hof- und Staatsdruckerei, 1912).

25 M. Baczkowski, *Pod czarno-żółtymi sztandarami*, 36; MSJ 1880, 1881,1882, 389.

26
*Eingaben*, 4.

27 J. Ch. Allmayer-Beck, ‘Die bewaffnete Macht in Staat und Gesellschaft’, 85–86.

28 “Gesetz vom 5. December 1868, womit für die im Reichsrathe vertretenen Königreich und Länder die Art Weise der Erfüllung der Wehrpflicht geregelt wird, *Reichsgesetzblatt*, 1868, LXVI,151, 438; ‘Gesetz vom 2. October 1882, womit mehrere Paragraphe des Wehrgesetzes vom 5. December 1868 (R.G.Bl. Nr. 151) abgeändert werden’, *Reichsgesetzblatt*, 1882, LVII, 153, 565; ‘*Ges*etz vom 11. April 1889, betreffend die Einführung eines neuen Wehrgesetzes’*, Reichsgesetzblatt*, 1889, XV, 41, 190–191; ‘*Ges*etz vom 5. Juli 1912, betreffend die Einführung eines neuen Wehrgesetzes’ *,Reichsgesetzblatt*, 1912, LIV,128, 412. Szerzej na temat powszechnej służby wojskowej w Austro-Węgrzech patrz: Ch. Ehrmann-Hämmerle, ‘Verhandelt und bestätigt – oder eben nicht? Gemeinden und Allgemeine Wehrpflicht in Österreich-Ungarn (1868–1914/18)’, *Geschichte und Region*, 2005, 14, 15–41; M. Weigl-Burnautzki, *Internierung und Militärdienst: die “Lösung der Zigeunerfrage” in Österreich-Ungarn im Ersten Weltkrieg* (Wien: Böhlau Verlag, 2022).

29 M. Baczkowski, *Pod czarno-żółtymi sztandarami*, 35.

30 W. Wagner, ‘Die k.(u.)k Armee – Gliederung und Aufgabenstellung’, in A. Wandruszka, P. Urbanitsch, eds, *Die Habsburgermonarchie 1848-1918.* Band V: *Die bewaffnete Macht*, (Wien: Verlag der Österreichischen Akademie der Wissenschaften, 1987), 395, Karte 2; M. Baczkowski, “Ilu Polaków walczyło w armii austro-węgierskiej podczas I wojny światowej*?*”, in A. Smoliński, eds, *Mało znana Wielka Wojna. Studia i szkice z dziejów I wojny światowej*, (Oświęcim: Napoleon V, 2014), 67.

31 W. Wagner, ‘Die k.(u.)k Armee – Gliederung und Aufgabenstellung’, 395, 398, Karte 2; *Verordnungsblatt für kaiserlich und königliche Heer. Normalverordnungsblatt (Jänner bis Ende Dezember 1889).* 1890, 24, 165.

32
*Instruktion zur ärztlichen Untersuchung der Wehrpflichtigen*, (Wien Die kaiserlich-königliche Hof- und Staatsdruckerei, 1869); *Instruktion zur ärztlichen Untersuchung der Wehrpflichtigen*, (Wien: Die kaiserlich-königliche Hof- und Staatsdruckerei, 1883); *Vorschrift für die ärztliche Untersuchung der Wehrpflichtigen*, (Wien: Der kaiserlich-königlichen Hof- und Staatsdruckerei, 1889); *Vorschrift für die ärztliche Untersuchung der Wehrpflichtigen*, (Wien: Die kaiserlich-königliche Hof- und Staatsdruckerei, 1912).

33
*Untersuchung*, Wien 1889, 10, 21.

34
*Vorschrift für die ärztliche Untersuchung der Wehrpflichtigen*, (Wien: Die kaiserlich-königliche Hof- und Staatsdruckerei, 1912), 9,11.

35 J. Scheibert, W. Porth, *Illustriertes Militär-Lexikon für die k. und k. österreichisch-ungarische und deutsche Armee*, (Berlin: Verlag von W. Pauli’s Nachf., 1897), 756-757; *Superarbitrungsvorschrift fur die Personen des k.k. Heeres*, (Wien: Die kaiserlich-königliche Hof- und Staatsdruckerei, 1885); *Verzeichnis der in Kraft stehenden und der in Bearbeitung befindlichen Dienstbücher und Vorschriften, als Anhang I zur Geschäftsordnung für das k.u.k. Heer,* (Wien: Die kaiserlich-königliche Hof- und Staatsdruckerei, 1912), 23.

36 SSRKLJ 1882, 46; SSRKLJ 1883, 46; SSRKLJ 1884, 62, 64; SSRKLJ 1885, 62, 64; SSRKLJ 1886, 62, 64; SSRKLJ 1887, 62, 64; SSRKLJ 1888, 64, 66; SSRKLJ 1889, 64, 66; SSRKLJ 1890, 66, 68; SSRKLJ 1891, 70, 72; SSRKLJ 1892, 70, 72; SSRKLJ 1893, 70, 72 SSRKLJ 1894, 70, 72; SSRKLJ 1895, 70, 72; SSRKLJ 1896, 70, 72; SSRKLJ 1897, 70, 72; SSRKLJ 1898, 70, 72; SSRKLJ 1899, 72, 74; SSRKLJ 1900, 72, 74; SSRKLJ 1901, 72, 74 SSRKLJ 1902, 72, 74; SSRKLJ 1903, 72, 74; SSRKLJ 1904, 72, 74; SSRKLJ 1905, 72, 74; *Bewegung der Bevölkerung der im Reichsrate vertretenen Königreiche und Länder im Jahre 1906*, (Wien: Die kaiserlich-königliche Hof- und Staatsdruckerei 1908) 72, 74 – further in the footnotes as BBRKLJ; SSRKLJ 1907, 1908, 1909, 1910, 72, 74, 174, 176, 274, 276, 383, 385; SSRKLJ 1911, 91, 93; SSRKLJ 1912, 91, 93; *Statistik des Sanitätswesens in Österreich für das Jahr 1913*, (Wien: Die kaiserlich-königliche Hof- und Staatsdruckerei 1917), 81, 83 – further in the footnotes as SÖJ; H. Fassmann, „Die Bevölkerungsentwicklung 1850-1910“, in H. Rumpler, P. Urbanitsch, eds, *Die Habsburgermonarchie 1848-1919*, Band IX: *Soziale Strukturen*, (Wien: Verlag der Österreichischen Akademie der Wissenschaften, 2010), 1. Teil, Teilband 2, 160.

37 BBRKLJ 1896, 2, 156-157; BBRKLJ 1897, 1, 156-157; BBRKLJ 1898, 3, 124-125; BBRKLJ 1899, 3, 126-127; BBRKLJ 1900, 1, 126-127; BBRKLJ 1910, 1, 122-123; BBRKLJ 1913, 1, 140-141; MSÉ 1898, 37; MSÉ 1899, 48; MSÉ 1900, 48; MSÉ 1901, 49; MSÉ 1902, 68; MSÉ 1903, 63; MSÉ 1904, 63; MSÉ 1905, 45; MSÉ 1906, 56; MSÉ 1907, 56; MSÉ 1908, 56; MSÉ 1909, 53; MSÉ 1910, 47; MSÉ 1911, 51; MSÉ 1913, 51; H. Fassmann, “Die Bevölkerungsentwicklung 1850-1910“, 160.

38 See fn 36.

39 MSJ 1880, 1881,1882, 344-345; MSJ 1883, 1884, 22-23, 194-195; MSJ, 1885, 230-231; MSJ 1886, 206-207; MSJ, 1887, 222-223; MSJ 1888, 212-213; MSJ 1889, 216-217; MSJ 1890, 220-221; MSJ 1891, 218-219; MSJ 1892, 226-227; MSJ 1893, 238-239; MSJ 1894, 274-275; SSMH 1895, 126-127; SSMH 1896, 128-129; SSMH 1897, 132-133; SSMH 1898, 130-131; SSMH 1899, 126-127; SSMH 1900, 126-127; SSMH 1901, 120-121; SSMH 1902, 114-115; SSMH 1903, 110-111; SSMH 1904, 110-111; SBH 1905, 110-111; SBH 1906, 108-109; SBH 1907, 106-107.

40
*Untersuchung*, Wien 1889, 21-39.

41 See footnote 37.

42 M. Pammer, “Die Sozialstrukturen im Spiegel der Sozialstatistik“, in H. Rumpler, P. Urbanitsch, eds, *Die Habsburgermonarchie 1848-1919*, Band IX: *Soziale Strukturen*, (Wien: Verlag der Österreichischen Akademie der Wissenschaften, 2010), 1. Teil, Teilband 2, 1583; BBRKLJ 1900, *1*, 126; BBRKLJ 1910, *1*, 122-123; MSÉ *1901,* 47; MSÉ 1911, 47; SSMH 1900, 135; SBH 1910, Wien 1911, 49.

43 H. Rumpler, M. Seger, Die Habsburgermonarchie 1848-1918, Band IX: *Soziale Strukturen*, (Wien: Verlag der Österreichischen Akademie der Wissenschaften, 2010), 2. Teilband, Kartenband, 267, 280, 282, 285, 287, 295-297.

44 MSJ 1900, 58,62,66; MSJ 1901, 58,62,66; MSJ 1902, 58,62,66; MSJ 1903, 58,62,66; MSJ 1904, 58,62,66; MSJ 1905, 58,62,66; MSJ 1906, 58,62,66; MSJ 1907, 58,62,66; MSJ 1908, 58,62,66; MSJ 1909, 58,62,66; MSJ 1910, 58,62,66.

45 W. Szczepanik, “Wzajemne relacje między wojskiem a społeczeństwem w galicyjskim garnizonie przełomu XIX i XX wieku na przykładzie Tarnowa”, *Prace Historyczne*, 2018, *145*, 810-817.

46 BBRKLJ 1896, 2, 142, 144, 148, 150, 154, 156-157; BBRKLJ 1897, 1, 142, 144, 148,150, 154, 156-157; BBRKLJ 1898, 3, 110, 112, 116, 118, 120, 122, 124-125; BBRKLJ 1899, 3, 112, 114, 118, 120, 122, 124, 126; BBRKLJ 1900, 1, 112, 114, 118, 120, 122, 126; BBRKLJ 1901, 1, 106, 108, 112, 114, 116, 120-121; BBRKLJ 1902, 3, 106, 108, 112, 114, 116, 120-121; BBRKLJ 1903, 1, 48, 50, 54, 56,58, 62-63; BBRKLJ 1904, 1, 106, 108, 112, 114, 116, 120-121; BBRKLJ 1905, 1, 50, 52, 56, 58, 60; BBRKLJ 1906, 1, 50, 52, 56, 58, 60, 64-65; BBRKLJ 1907, 1, 106, 108, 112, 114, 116, 120-121; BBRKLJ 1908, 1909, 3, 50, 52, 54, 56, 60, 62-63, 150, 152, 154, 156, 160, 162-163; BBRKLJ 1910, 1, 110, 112, 114, 116, 120, 122-123 BBRKLJ 1911, 1, 60, 62, 64, 66, 68, 70, 72; BBRKLJ 1913, 1, 126, 128, 130, 132, 134, 136, 138, 140-141.

47 BBRKLJ *1896*, 2, 158-159, 164-165, 170-171, 174-176; BBRKLJ *1897*, 1, 158-159, 164-165, 170-171, 174-176; BBRKLJ *1898*, 3, 126-127, 132-133, 138-139, 142-143; BBRKLJ *1899*, 3, 128-129, 134-135, 140-141,144-146; BBRKLJ *1900*, 1, 128-129, 124-135, 140-141,144-146; BBRKLJ *1901*, 1, 122-123, 128-129, 134-135, 138-140; BBRKLJ *1902*, 3, 122-123, 128-129, 134-135, 138-139, 1; BBRKLJ *1904*, 1, 122-123, 128-129, 134-135, 138-140; BBRKLJ *1907*, 1, 122-123, 128-129, 134-135, 138-139; BBRKLJ *1910*, 1, 124-125, 130-131,136-137,140-142; *Bewegung der Bevölkerung Österreichs im Jahre 1913*, (Wien: Die kaiserlich-königliche Hof- und Staatsdruckerei, 1918), *1*, 142-143, 148-149, 154-155, 156-157, 160.

48 MSJ 1886, 210; MSJ 1893, 242.

49 M. Ch. Ortner, *Organisationshanbuch der k.u.k Armee im Ersten Weltkrieg 1914-1918. Heeresstruktur, Aufbau und Gliederung*, (Wien: Militaria Verlag, 2018), 286-287; *Eingaben*, 4.

50 M. Ch. Ortner, *Organisationshanbuch*, 288.

51 This was isolated in 1944.

52
*Militärpharmakopöe*, (Wien: Die kaiserlich-königliche Hof- und Staatsdruckerei, 1891), 181-182.

53 T. Dormandy, *The White Death*, 46-47.

54 Ibid., 267.

55 T. G. Benedek, “Ehrlich’s Theory of drug Action and anti-Tuberculous Gold Therapy“, *Pharmacy in History*, 2005, *47*, 18-22; “The History of Gold Therapy for Tuberculosis“, *Journal of the History of Medicine and Allied Sciences*, 2004, *59*, 50-89.

56
*Militärpharmakopöe*.

57 MSJ 1885, 187-189.

58 T. Dormandy, *The White Death*, 352-353.; S. M. Tuli, “Historical aspects of Pott’s disease (spinal tuberculosis) management”, *European Spine Journal*, 2013, 22, 529-538;. E. Dietrich-Daum, Die “Wiener Krankheit“, 195.

59 MSJ 1883, 1884, 350-354; MSJ 1885, 380-395; MSJ 1886, 366-371; MSJ 1887, 382-387; MSJ 1888, 372-381; MSJ 1889, 376-385; MSJ 1890, 380-391; MSJ 1891, 378-389; MSJ 1892, 386-397; MSJ 1893, 398-407; MSJ 1894, 400-407; SSMH 1895, 270-277; SSMH 1896, 272-280; SSMH 1897, 278-289; SSMH 1898, 274-287; SSMH 1899, 270-278; SSMH 1900, 270-278; SSMH 1901, 264-274; SSMH 1902, 258-267; SSMH 1903, 254-264; SSMH 1904, 254-264; SBH 1905, 254-261; SBH 1906, 252-259; SBH 1907, 250-257; SBH 1908, 69-70; SBH 1909, 91-95; SBH 1910, 93-97; SBH 1911, 93-97; SBH 1912, 101-112.

60
J. A. Odell, Surgery“; E. Dietrich-Daum, *Die “Wiener Krankheit“*, 210-211.

61 See fn. 58.

62 SSMH 1897, 283.

63
*Untersuchung*, Wien 1889 21, 33; *Untersuchung*, Wien 1912 27, 33, 39, 41.

64
*Vorschrift*, Wien 1885, 71-74.

65 Soldiers were sent on leave for no longer than three months. For TB purposes, mere leave for treatment reasons could not produce any positive effects as prior to the discovery of streptomycin there was no possibility to treat tuberculosis effectively. MSJ 1880, 1881,1882, 469, 475;; MSJ 1883, 1884, 151, 157, 330, 338; MSJ, 1885, 370, 378; MSJ 1886, 346, 354; MSJ 1887, 362, 370; MSJ 1888, 352, 360; MSJ 1889, 356, 364; MSJ 1890, 360, 368; MSJ 1891, 358, 366; MSJ 1892, 366, 374; MSJ 1893, 378, 386; MSJ 1894, 358, 366.

66
*Sanitätbericht über das Reichsheer für das Jahre 1933 (1 Januar bis 30 Dezember)*, *1934 (1 Januar bis 30 Dezember)* und für 1935 (für die Zeit vom 1. Januar bis 30 September 1935) (Berlin: E.S. Mittler &Sohn, 1940), 15.

67 MSJ 1894, 274.

68
*Untersuchung*, Wien 1912, 9.

69 Clemens Peter von Pirquet (in 1907), Charles Mantoux (in 1908) and Ernst Moro (in 1909) developed tuberculin-based tests to detect inactive disease. However, these advances were very slow to spread (they became more common in the late 1920s). The low accuracy of the tests (pure tuberculin was not obtained by Florence Seibert until 1931) and the lack of effective anti-tuberculosis drugs, which meant that the diagnosis of inactive tuberculosis could not be followed up with therapy, were responsible for this. S. H. E. Kaufmann, E. Rubin, eds, *Handbook of Tuberculosis. Molecular Biology and Biochemistry,* (Weinheim: Wiley-VCH Verlag GmbH & Co, 2008), XXVI; T. Dormandy, *The White Death*, 142, 206; E. Dietrich-Daum, *Die “Wiener Krankheit“,* 184-189; OeStA KA ZSt KM HR Akten 4900, 43-8.

70 OeStA KA ZSt KM HR Akten 6764, Abt. 14 Nr. 5987, v. 1913; *Sanitätbericht über das Reichsheer für das Jahr 1930 (1 Januar bis 30 Dezember)*, (Berlin: E.S. Mittler &Sohn, 1933), 13-14.

71 M. Baczkowski*, Pod czarno-żółtymi sztandarami*, 142-144; J. Ch. Allmayer-Beck, „Die bewaffnete Macht in Staat und Gesellschaft“, 113-114.

72 OeStA KA ZSt KM HR Akten 3213, *Instruktion für die Unterricht über die Gesundheitspflege zum Gebrauche in den Unteroffizieres- und Manschaftsschulen*, (Wien: Die kaiserlich-königliche Hof- und Staatsdruckerei, 1881), 15-16; *Instruktion für den Unterricht über die Gesundheitspflege zum Gebrauche in den Unteroffizieres- und Manschaftsschulen*, (Wien: Die kaiserlich-königliche Hof- und Staatsdruckerei, 1894), 17-18; OeStA AVA Inneres MdI SA A 3153, *Gesundheits – Regeln*.

73
*Instruktion für die Unterricht über die Gesundheitspflege zum Gebrauche in den Unteroffizieres- und Manschaftsschulen*, (Wien: Die kaiserlich-königliche Hof- und Staatsdruckerei, 1909), 41-42.

74 I. Munk, J. Uffelmann, C. A. Ewald, *Ernährung des gesunden und kranken Menschen. Handbuch der Diätetik für Ärzte, Verwaltungsbeamte und Vorsteher von Heil- und Pflege-Anstalten*, (Wien: Urban & Schwarzenberg, 1895), 212-213; *L-2 Vorschrift für die Verpflegung im Kriege,* (Wien: Die kaiserlich-königliche Hof- und Staatsdruckerei, 1909), 2; P. Franciszek, „Dieta chłopów galicyjskich na przełomie XIX i XX wieku”, in *Rocznik dziejów społecznych i gospodarczych*, 2016, LXXVI, 310.

