# Supplementary material for: A White Death Among the Ranks. Tuberculosis in the Austro-Hungarian Army, 1882–1914
Source: Soc Hist Med. 2025 May 12;39(1):48–70. doi: 10.1093/shm/hkae095 (PMC13034125; doi:10.1093/shm/hkae095)
Supplement: hkae095_suppl_Supplementary_Tables_S1 [file hkae095_suppl_supplementary_tables_s1.docx]

| **Table II**  **TB patients in 1882-1912 per 1 000 soldiers of the Imperial-Royal Armies.** | | | | | | | | | | | |
| --- | --- | --- | --- | --- | --- | --- | --- | --- | --- | --- | --- |
| **Year** | **Infantry** | **Light infantry (Jäger)** | **Cavalry** | **Field artillery** | **Fort artillery** | **Artillery technical divisions*** | **Pioneer units** | **Healthcare unit** | **Rolling stock** | **Mean for the Common Army** | **Number of the Common Army**** |
| 1882 | 6,2 | 6 | 6,3 | 6,2 | 5,6 | 8,2 | 4,6 | 8,8 | 7,5 | **6,3** | 276 191 |
| 1883 | 6,9 | 5,6 | 8,4 | 7,3 | 6,2 | 7,6 | 8,4 | 6,9 | 6 | **7** | 267 286 |
| 1884 | 5,7 | 4,9 | 8 | 4,4 | 6,1 | 5,6 | 6,9 | 6,9 | 6,3 | **5,9** | 266 780 |
| 1885 | 5,7 | 4,9 | 4,8 | 5 | 4,9 | 5 | 4,5 | 13,5 | 9,3 | **5,8** | 265 173 |
| 1886 | 5,7 | 5,3 | 6,5 | 7,2 | 5,9 | 6,2 | 8 | 12,2 | 4,2 | **6** | 268 667 |
| 1887 | 5 | 4 | 6 | 7,2 | 6,7 | 4,4 | 4,4 | 10,7 | 7,6 | **5,4** | 272 223 |
| 1888 | 5,5 | 4,9 | 6,5 | 8 | 4,2 | 5,3 | 1,5 | 12,8 | 4,7 | **5,7** | 279 825 |
| 1889 | 5,8 | 4 | 6,1 | 6,3 | 5,3 | 10,3 | 5,1 | 9,6 | 7,2 | **5,8** | 281 897 |
| 1890 | 6 | 4,6 | 2,7 | 6,6 | 5,9 | 5,8 | 3,7 | 8,6 | 8 | **6,3** | 281 112 |
| 1891 | 5,8 | 4,6 | 7,1 | 6,4 | 6,3 | 6,1 | 3,2 | 7,7 | 4,8 | **5,8** | 284 828 |
| 1892 | 5 | 4,1 | 6 | 5,5 | 4,6 | 5,9 | 3,9 | 6,5 | 5,6 | **5,2** | 287 885 |
| 1893 | 4,9 | 5,5 | 5,6 | 5,5 | 5,5 | 0 | 4 | 4,9 | 5,1 | **5** | 300 000 |
| 1894 | 3,1 | 2,7 | 5 | 4,4 | 2,7 | 1,7 | 2,6 | 6,3 | 1,8 | **3,5** | 280 858 |
| 1895 | 2,8 | 3,1 | 4,2 | 3,2 | 3,7 | 3,6 | 2 | 4,3 | 1,5 | **3** | 288 000 |
| 1896 | 2,2 | 3 | 3,4 | 3,3 | 2,6 | 1,8 | 3,3 | 7,6 | 1,4 | **2,8** | 281 072 |
| 1897 | 2,4 | 2,5 | 3,9 | 3,3 | 1,6 | 2,8 | 4,3 | 4,7 | 4,7 | **2,9** | 282 759 |
| 1898 | 2,3 | 2,4 | 3,1 | 2,7 | 1,6 | 1,8 | 5,3 | 6,5 | 3,6 | **2,8** | 282 500 |
| 1899 | 2,3 | 2,2 | 2,5 | 2,6 | 2,1 | 1,2 | 4,4 | 5,5 | 4,8 | **2,6** | 287 308 |
| 1900 | 2,6 | 2,4 | 3 | 3 | 1,8 | 1,1 | 4,5 | 3,1 | 3,5 | **2,8** | 288 929 |
| 1901 | 2,2 | 2,2 | 2,8 | 2,1 | 2,8 | 4,5 | 4,8 | 4,7 | 2,5 | **2,5** | 296 800 |
| 1902 | 2,5 | 1,9 | 3,8 | 4 | 3,5 | 2,1 | 5,6 | 5,8 | 3,7 | **3,1** | 289 033 |
| 1903 | 2,6 | 2,5 | 3,4 | 4,2 | 2,8 | 4,3 | 4,8 | 3,7 | 8,1 | **3,1** | 285 162 |
| 1904 | 2,7 | 2,2 | 3,1 | 2,9 | 2,1 | 0 | 3,2 | 3 | 6,4 | **2,9** | 284 138 |
| 1905 | 3,9 | 3 | 5 | 4,9 | 3,1 | 8,4 | 4,8 | 4,3 | 8,2 | **4,3** | 279 535 |
| 1906 | 3,3 | 2,9 | 4,3 | 3,2 | 2,8 | 6,5 | 2,3 | 3,3 | 7,7 | **3,6** | 273 334 |
| 1907 | 3,3 | 2,9 | 4,3 | 3,2 | 2,8 | 6,5 | 2,3 | 3,3 | 7,7 | **3,6** | 287 223 |
| 1908 | 4 | 3,3 | 5,2 | 4,3 | 2,8 | 5,4 | 4,8 | 7,1 | 4 | **4,2** | 385 502 |
| 1909 | 4,4 | 3 | 5,9 | 4,3 | 3,5 | 4,1 | 3,7 | 5,1 | 4,5 | **4,5** | 358 645 |
| 1910 | 4,2 | 3,7 | 5,2 | 5 | 4,1 | 3,9 | 4,7 | 5,8 | 5,7 | **4,5** | 361 019 |
| 1911 | 4,3 | 5 | 5,5 | 5,3 | 3,8 | 5,2 | 4,4 | 5,9 | 6,6 | **4,7** | 359 628 |
| 1912 | 4,2 | 3 | 4,6 | 3,8 | 2,8 | 3 | 4,3 | 7 | 3,7 | **4,1** | 392 483 |
| Source: MSJ 1880, 1881,1882, 389; MSJ 1883, 1884, 63, 235; MSJ, 1885, 275; MSJ 1886, 251; MSJ, 1887, 267; MSJ 1888, 257; MSJ 1889, 261; MSJ 1890, 265; MSJ 1891, 263; MSJ 1892, 271; MSJ 1893, 283; MSJ 1894, 283; SSMH 1895, 137; SSMH 1896, 129; SSMH 1897, 141; SSMH 1898, 139; SSMH 1899, 135; SSMH 1900, 135; SSMH 1901, 129; SSMH 1902, 123; SSMH 1903, 119; SSMH 1904, 119; SSMH 1905, 119; SBH 1906, 109; SBH 1907, 115; SBH 1908, 47 SBH 1909, 47; SBH 1910, 49; SBH 1911, 49; SBH 1912, 55; MSJ 1908, 143, 145; MSJ 1909, 143, 145; MSJ 1910, 143, 145; MSJ 1911, 143, 145-146; MSJ 157, 159-160.  *Until 1892 Gene-truppen,  **The average yearly quantity of the joint army between 1882 and 1907 was calculated using the relative and absolute numbers of tuberculosis patients in the joint army. The figures thus obtained differ, sometimes significantly, from the reported state of boarding by the statistical yearbooks. The reasons for this are difficult to establish, as raised by Michał Baczkowski. | | | | | | | | | | | |
